# Outcomes and financial comparison of percutaneous debulking versus surgical management of tricuspid endocarditis

**DOI:** 10.1186/s13019-026-04061-5

**Published:** 2026-05-04

**Authors:** Sanjhai L. Ramdeen, Sahaj S. Shah, Clement J. Rajakumar, Jordan G. Law, Kent A. Strohecker, Stephanie A. Buczkowski, Damian D. Mason, Michael E. Friscia, Michael A. Bresticker, Shikhar Agarwal, Carlo R. Bartoli

**Affiliations:** 1 Department of General Surgery, Geisinger Medical Center, Danville, PA USA; 2 Geisinger Commonwealth School of Medicine, Scranton, PA USA; 3 Phenomic Analytics and Clinical Data Core, Geisinger Health System, Danville, PA USA; 4 Department of Revenue Management, Geisinger Health System, Danville, PA USA; 5 Department of Research, Geisinger Medical Center, Danville, PA USA; 6 Division of Cardiothoracic Surgery, Geisinger Medical Center, Danville, PA USA; 7 Division of Cardiology, Geisinger Medical Center, Danville, PA USA; 8 Division of Pediatric and Adult Congenital Cardiothoracic Surgery, Geisinger Medical Center, Danville, PA USA; 9 Pediatric and Adult Congenital Cardiothoracic Surgery, Geisinger Medical Center, 100 North Academy Ave, Danville, PA 19821 USA

**Keywords:** Intravenous drug abuse, Endocarditis, AngioVac, Percutaneous debulking, Vegectomy, Tricuspid valve repair, Replacement

## Abstract

**Background:**

Intravenous drug abuse (IVDA) has increased the incidence of infective endocarditis. Standard management includes traditional open surgery and more recently described percutaneous tricuspid valve debulking. Study goals were to compare clinical outcomes and identify financial differences between percutaneous tricuspid debulking and tricuspid surgery for isolated tricuspid valve endocarditis.

**Methods:**

A single-center, retrospective cohort patient study of isolated tricuspid valve endocarditis was performed. Patients underwent either percutaneous debulking with the AngioVac system (n=14, 83% IVDA) or tricuspid valve surgery (n=23, 76% IVDA). Length of stay, readmission rates, mortality, echocardiographic parameters, hematologic markers, transfusion rates, and total charges for index hospitalization were evaluated between groups.

**Results:**

In patients who underwent either percutaneous debulking or open surgery, length of stay (17±17 vs 20±13 days, p=0.48), 30-day readmission (29% vs 26%, p=0.87), in-hospital mortality (7% vs 0%, p=0.20), and 30-day mortality (7% vs 0%, p=0.20) were not statistically different. One-year mortality (21% vs 4%, p=0.11) trended toward but did not reach significance. Postoperative tricuspid valve regurgitation (2.5±1.1 vs 1.0±0.3, p<0.0001) and transfusion rates (2±3 vs 6±6 units, p=0.02) were significantly different between therapies. Total charges for hospitalization were not statistically different ($557,066±457,520 vs $571,615±324,254, p=0.91).

**Conclusions:**

Tricuspid debulking is a potential alternative to surgery for patients with infective tricuspid endocarditis. Similar outcomes, costs, and avoidance of prosthetic material in patients with active IVDA are potential benefits.

**Graphical Abstract:**

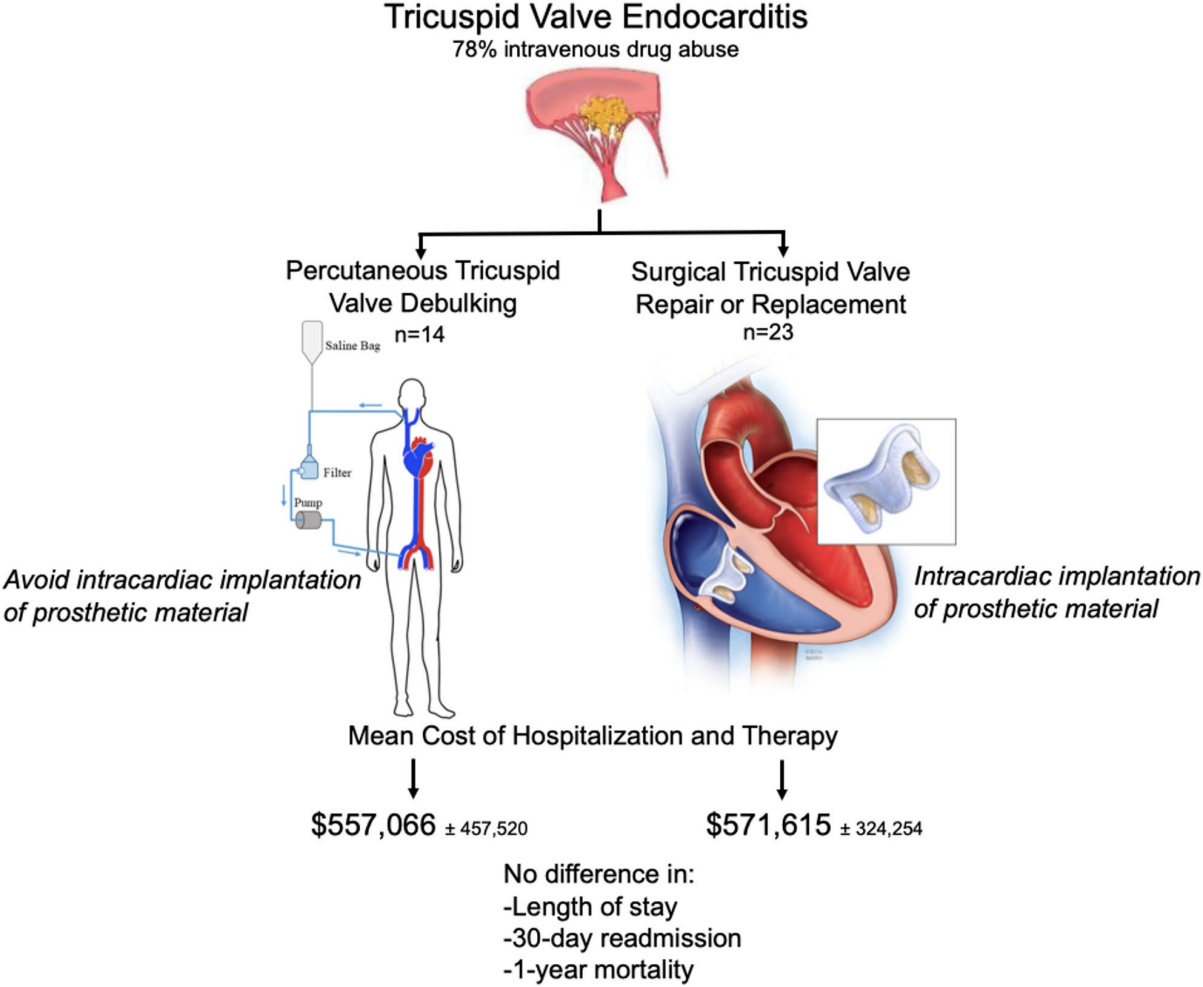

## Introduction

Infective endocarditis has increased over the past decade [1–5]. This is in large part due to current rates of intravenous drug abuse (IVDA) that cost the United States billions of dollars [1, 2]. Mortality rates in patients less than 35 years of age have risen significantly over two decades [6, 7].

Recently, percutaneous debulking of vegetation from the tricuspid valve has been reported in patients with infective endocarditis [8–19]. After venous femoral cannulation, the AngioVac system (AngioDynamics, Latham, NY), which was designed to remove clot from the heart [8, 9, 20–22], debulks and removes infected tissue from the right heart. Survival rates of 85 to 100% are reported [8, 11, 23, 24]. However, relative safety, outcomes, and costs are not well studied. Potential medical benefits include avoidance of sternotomy, cardiopulmonary bypass, and implantation of prosthetic material in a bacteremic patient who uses intravenous drugs. Additional medical benefits may include faster resolution of bacteremia, shorter intensive care unit and hospital stay, and fewer transfusions [10, 12, 24].

It is unclear if percutaneous tricuspid debulking improves outcomes or provides financial benefit over tricuspid valve surgery. The major goal of this study was to evaluate and compare patient outcomes and charges for percutaneous debulking versus surgery in patients with tricuspid valve endocarditis.

## Methods

### Study design

Geisinger Institutional Review Board (IRB) approval was obtained (IRB 2021 − 0970) to perform a retrospective, cohort study. Given the retrospective nature of the study, the IRB waived individual patient informed consent. Patients studied received care between March 2016 and March 2022. Consecutive AngioVac patients (*n* = 14) were studied. A similar cohort of isolated tricuspid valve surgery patients (*n* = 23) were queried for comparison.

## Clinical decision making

A multidisciplinary team of cardiac surgery, interventional cardiology, general cardiology, and critical care considered patients with an indication for tricuspid valve surgery for percutaneous debulking. Patient age, history of IVDA, active recent use of intravenous drugs, anticipated or stated intention of recidivism, history of prior endocarditis, history of prior heart surgery, end-organ function, presence of bacteremia, active sepsis, infectious organism, and surgical risk were considered on a case-by-case basis. In general, older and sicker patients with greater surgical risk were deemed possibly more suitable for percutaneous debulking.

### AngioVac procedures

The AngioVac system (AngioDynamics, Latham, NY) is a percutaneous aspiration circuit designed to remove clot from systemic veins and the right heart [20]. Percutaneous debulking of infected vegetation from the tricuspid valve was performed per vendor instructions and Geisinger clinical practice. The right internal jugular vein was cannulated percutaneously with an inflow trumpet that aspirates blood. Inflow was connected to a centrifugal pump with a filter. A femoral vein was cannulated and connected to the pump for reinfusion of blood. During AngioVac therapy, the circuit aspirated, filtered, and reinfused 4 to 5 L/min of venous blood. With this system, we and others have removed thrombus, emboli, and vegetation from the heart [8–19].

### Data query

Data managers queried Geisinger Phenomic Analytics and Clinical Data Core database, which is a compilation of Epic electronic medical records, patient imaging, and billing data. Patient inclusion criteria were ≥18 years of age, isolated tricuspid valve endocarditis, percutaneous tricuspid valve debulking, or tricuspid valve surgery (repair or replacement). Exclusion criteria were multivalvular endocarditis, concomitant cardiac or valvular surgery at the time of tricuspid surgery, and/or subsequent cardiac surgery during the same hospitalization. Inpatient, outpatient, and emergency department visits, and patient problem lists were queried for infective endocarditis International Classification of Disease codes (revision 10). Surgical patients were included according to Current Procedural Terminology codes for tricuspid valve repair or replacement. IVDA was queried directly from Epic.

Patient charts and operative reports were reviewed and adjudicated. Patient demographics, length of stay, readmission, mortality, echocardiographic parameters, and hematologic markers were obtained from the electronic medical record. Perioperative transfusion data were obtained from the Geisinger blood bank.

Preoperative and postoperative echocardiographic reports were reviewed. An echocardiographic scoring system was used to classify right ventricular size (0 normal, 1 dilated), right ventricular function (0 normal, 1 mild reduction, 2 moderate reduction, 3 severe reduction), and tricuspid regurgitation (0 none, 1 mild, 2 moderate, 3 severe).

Financial data were obtained directly from the Geisinger Department of Revenue Management through a practice consultant. The sum of all charges from each patient during hospitalization that included preoperative, procedural, and postoperative charges represented total cost of hospitalization. Hospital charges included level of care, procedural and surgical charges, anesthesia charges, imaging studies, laboratory tests, medications, medical supplies, and consultations with specialists. Outpatient medical costs were not included.

### Statistical analysis

GraphPad, version 6.0 (Prism, La Jolla, CA) was used to perform statistical analyses. Kolmogorov-Smirnov tests determined data normality. Unpaired, two-way Student’s t-tests were performed for parametric data. Wilcoxon signed rank tests compared nonparametric data. A p-value < 0.05 (95% confidence) was considered statistically significant. Data are presented as mean±standard deviation.

## Results

### Patients

Patient demographics and co-morbid conditions were not statistically different between groups (Table   1). IVDA was the major cause of tricuspid endocarditis in both groups (AngioVac patients *n* = 14, 83% IVDA; surgical patients *n* = 23, 76% IVDA, repair *n* = 15, replacement *n* = 8, redo sternotomy *n* = 3). Surgical repair patients underwent either leaflet debridement and repair only (*n* = 3), leaflet debridement, repair, and extension with annuloplasty ring (*n* = 8), or leaflet debridement, repair, and extension with annuloplasty ring and neocords (*n* = 4). All patients who underwent tricuspid valve replacement received a bioprosthetic valve. All tricuspid valve repair or replacement operations were performed via midline sternotomy or redo midline sternotomy. Two patients with recurrent tricuspid endocarditis underwent two separate percutaneous debulking admissions. One patient with recurrent tricuspid endocarditis and IVDA underwent percutaneous debulking during a first hospitalization and tricuspid valve replacement during a subsequent hospitalization. Two patients were discharged on the same day after percutaneous tricuspid debulking.

**Table 1:** Patient demographics

	Percutaneous debulking			Tricuspid surgery		*p*-value
	*n* = 14			*n* = 23		
Age	36 ± 14			31 ± 7		0.17
Gender	43% Male	57% Female		65% Male	35% Female	0.43
IVDA*	83% Yes	17% No		76% Yes	26% No	0.87
Replacement or Repair	--			35% Replacement	65% Repair	--
Redo	--			13%		--
Total Hospital Cost	$557,066 ± 457,520			$571,615 ± 324,254		0.91

*IVDA, intravenous drug abuse

### Length of stay, readmission, and mortality

Hospital days before intervention were significantly lower for percutaneous debulking versus surgery (5 ± 4 vs. 9 ± 6 days, *p* = 0.02). Length of stay (17 ± 17 vs. 20 ± 13 days, *p* = 0.48), 30-day readmission (29% vs. 26%, *p* = 0.87), in-hospital mortality (7% vs. 0%, *p* = 0.20), and 30-day mortality (7% vs. 0%, *p* = 0.20) were not statistically different between groups (Table 2). One-year mortality (21% vs. 4%, *p* = 0.11) was higher in the AngioVac group and trended towards significance.

**Table 2:** Patient outcomes

	Percutaneous debulking		Tricuspid surgery		*p*-value
	*n* = 14		*n* = 23		
Length of Stay (days)	17 ± 17		20 ± 13		0.48
30-Day Readmission	29%		26%		0.87
In-Hospital Mortality	7%		0		0.20
30-Day Mortality	7%		0		0.20
1-Year Mortality	21%		4%		0.11

### Echocardiographic findings

There was no significant difference in preprocedural tricuspid regurgitation (2.2 ± 1.3 vs. 2.6 ± 0.8, *p* = 0.24). Postprocedural regurgitation was significantly greater in patients who underwent percutaneous debulking versus surgery (2.5 ± 1.1 vs. 1.0 ± 0.3, *p* < 0.0001). Postoperative left and right ventricular function were not different between groups. Right ventricular size trended toward statistical significance between groups (0.7 ± 0.5 vs. 0.4 ± 0.5, *p* = 0.09) (Table 3).

**Table 3:** Echocardiographic findings

	Percutaneous debulking	Tricuspid surgery	*p*-value
	*n* = 14	*n* = 23	
Preoperative Echocardiogram			
LVEF* (%)	56 ± 9	55 ± 6	0.78
RV† Size Score (0, 1)	0.8 ± 0.5	0.6 ± 0.5	0.42
RV† Function Score (0–3)	0.6 ± 0.9	0.2 ± 0.4	0.07
TR‡ Score (0–3)	2.2 ± 1.3	2.6 ± 0.8	0.24
Postoperative Echocardiogram			
LVEF* (%)	50 ± 13	53 ± 8	0.52
RV† Size Score (0, 1)	0.7 ± 0.5	0.4 ± 0.5	0.09
RV† Function Score (0–3)	1.0 ± 1.1	0.5 ± 0.8	0.15
TR‡ Score (0–3)	2.5 ± 1.1	1.0 ± 0.3	< 0.01

*LVEF, left ventricular ejection fraction; †RV, right ventricle; ‡TR, tricuspid regurgitation

### Blood product transfusion

Patients that underwent percutaneous debulking received significantly fewer blood products than those that underwent tricuspid surgery (2 ± 3 vs. 6 ± 6 units, *p* = 0.02). The surgical group received significantly more units of erythrocytes, plasma, cryoprecipitate, and total blood product units, all *p* < 0.05 (Table 4). Postoperative hemoglobin was significantly lower in patients that underwent percutaneous tricuspid valve debulking than tricuspid valve surgery (8.0 ± 0.6 vs. 9.7 ± 1.7 g/dL, *p* = 0.004).

**Table 4:** Blood product utilization

	Percutaneous debulking	Tricuspid surgery	*p*-value
	*n* = 14	*n* = 23	
Whole Blood (units)	0.1 ± 0.3	0 ± 0	0.20
Erythrocytes (units)	1.4 ± 2.3	3.7 ± 3.4	0.04
Plasma (units)	0.3 ± 0.7	1.7 ± 2.6	0.05
Platelets (units)	0.3 ± 0.7	0.6 ± 0.8	0.29
Cryoprecipitate (units)	0 ± 0	0.4 ± 0.7	0.05
Total Blood Product (units)	2.1 ± 3.3	6.3 ± 6.2	0.02

### Hospital charges

**Fig. 1:**
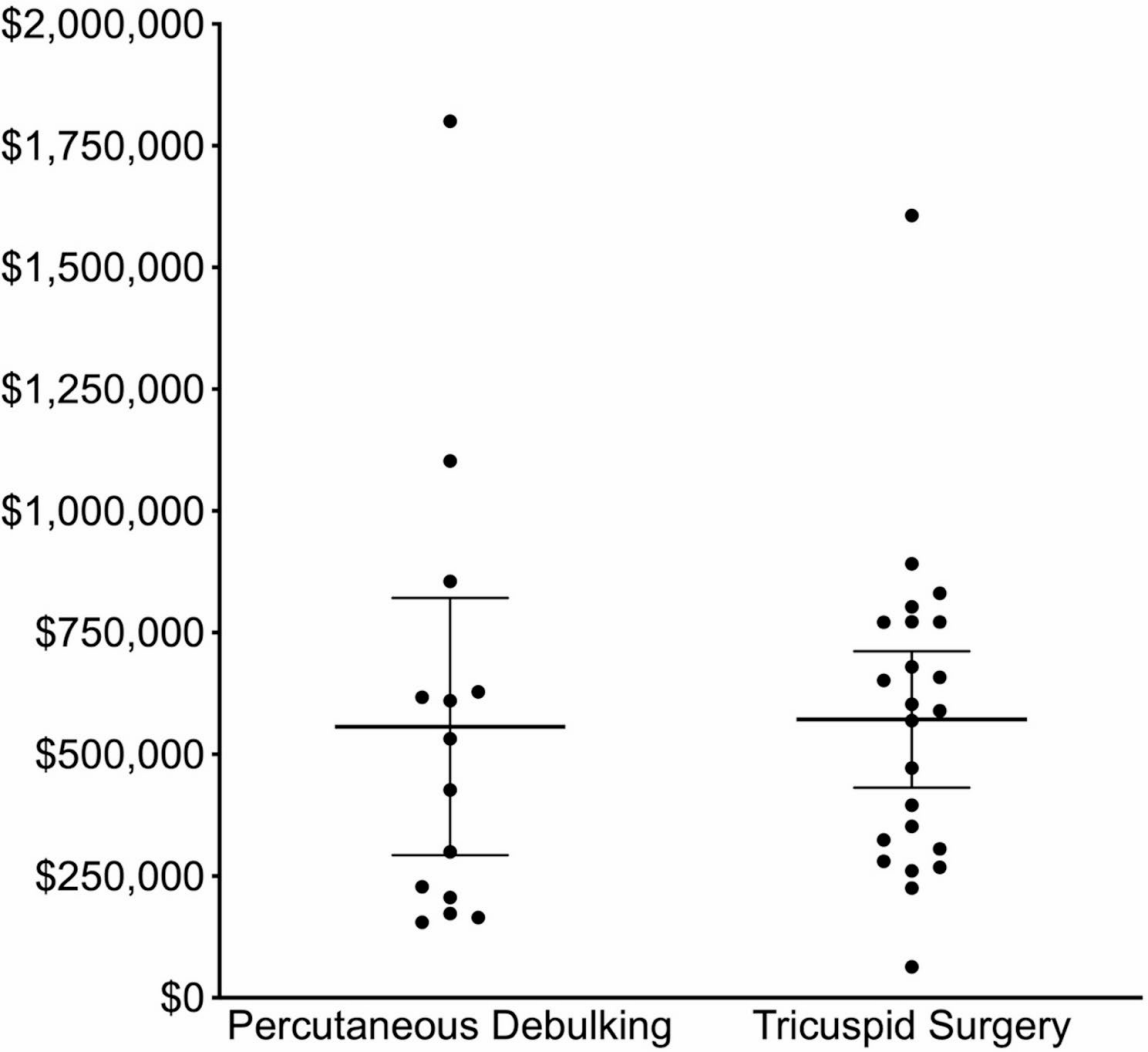
A dot plot with mean ±95% for total hospital costs demonstrates no significant financial difference between percutaneous debulking and tricuspid valve surgery in patients with isolated tricuspid valve endocarditis

Total charges for hospitalization were not statistically different between patients that underwent percutaneous debulking versus tricuspid surgery ($557,066 ± 457,520 vs. $571,615 ± 324,254, *p* = 0.91, Fig.1).

## Discussion

We evaluated costs and outcomes in patients with isolated tricuspid endocarditis who underwent either percutaneous debulking or isolated tricuspid valve surgery. Four principal findings were observed. First, there was no difference in length of stay, readmission, or short-term mortality between groups. Notably, there was a non-significant trend toward higher one-year mortality in patients that underwent percutaneous debulking. This finding may suggest limited durability of percutaneous debulking. Second, percutaneous debulking caused significant tricuspid regurgitation. This finding may limit durability, and sequelae of severe tricuspid insufficiency may have contributed to the observed difference in long-term mortality between therapies. Third, percutaneous debulking patients received fewer postprocedural blood transfusions, and postprocedural hemoglobin was lower. Fourth, the average total charge for hospitalization was greater than $550,000 per patient in both groups. There was no observed significant financial benefit between groups (Fig. 1).

### Epidemiology of IVDA-Associated infective endocarditis

IVDA-associated infective endocarditis causes significant financial burden to the United States healthcare system [1, 2, 25]. Patients with endocarditis and IVDA require longer hospital stays and higher financial costs than patients without IVDA [26, 27]. Surgical intervention more than doubles the median length of stay and greatly increases overall admission charges [26]. Unfortunately, 70% of patients continue IVDA after a first endocarditis operation, which commonly causes recurrent endocarditis [28]. Given the high rate of recidivism and endocarditis recurrence rate [29, 30], the 2015 European Society of Cardiology Guidelines recommended avoidance of surgery in patients with right-sided native-valve IVDA-associated infective endocarditis [31].

In 2009, the AngioVac system was approved by the Food and Drug Administration for extraction of central venous and right heart thrombus and emboli [8, 9, 20–22]. More recently, the AngioVac system has been used to avoid surgery by debulking infected tissue from the tricuspid valve [8–19], mitral valve [32–34], and pacemaker leads [13, 23, 35–37]. Debulking was successful in 61 to 98% of patients [8, 11, 13–15]. Survival rates of 85 to 100%^8,11,13,24^ are noteworthy for these complex patients. Potential complications included vascular access bleeding or infection, septic pulmonary embolization, pericardial effusion, tamponade, blood transfusion, worsened tricuspid regurgitation, renal failure, and intraoperative mortality [5, 9, 12, 14, 24]. Risks are counterbalanced by potential benefits of avoidance of sternotomy, cardiopulmonary bypass, and intracardiac implantation of prosthetic material in bacteremic patients who may resume intravenous drug use.

### Clinical decision making

Multidisciplinary decision making facilitated selection of patients that were appropriate for tricuspid valve debulking. Repeated percutaneous debulking procedures were possible in the same patient, and percutaneous debulking did not preclude later tricuspid valve surgery. Further clinical study is needed to refine patient selection to optimize outcomes.

### Avoidance of prosthetic material in patients with active IVDA

Intracardiac prosthetic material is a nidus for endocarditis. Tricuspid valvectomy is well described in patients that require tricuspid surgery in whom postoperative IVDA is anticipated [38]. In these patients, the tricuspid valve is removed entirely, and implantation of prosthetic material is avoided. Patients tolerate significant tricuspid regurgitation for years [38]. In patients who cease IVDA, interval redo sternotomy and tricuspid valve replacement may be performed. Similarly, we observed that percutaneous debulking avoided intracardiac implantation of prosthetic material and worsened tricuspid regurgitation. This was also well tolerated in our patients, two of whom later underwent first-time sternotomy and tricuspid valve replacement after successful cessation of IVDA.

### Financial implications

Percutaneous debulking was not associated with clear financial benefit versus tricuspid surgery. With either therapy, the average charges to hospitalize and manage tricuspid endocarditis was nearly $600,000 per patient. This financial burden should be examined within the broader context of contemporary health care delivery. Strategies to address addiction may be an important factor to prevent recurrence [39, 40]. Financial, social, and ethical implications of spending approximately $550,000 to treat patients with high recidivism and frequent recurrence may be considered in the context of contemporary health care delivery and medical economics.

Outpatient percutaneous tricuspid valve debulking was performed. Two patients were discharged on the same day. Younger patients with subclinical endocarditis may be amenable to outpatient management. This observation partially accounted for significantly fewer hospital days prior to intervention (5 vs. 9 inpatient days, *p* = 0.02) and relative differences in management between interventional and surgical groups.

Postoperative hemoglobin and transfusion were significantly lower in tricuspid debulking patients. Patient blood volume is diluted with crystalloid prime from the AngioVac circuit. This finding likely also reflects other differences in postcardiotomy management versus a transcatheter approach.

### Limitations

Retrospective, single-center studies are inherently limited. Findings should be interpreted within the context of a study that was not specifically designed to compare percutaneous debulking versus tricuspid surgery. The decision to offer percutaneous debulking was not randomized but was made on a case-by-case basis. As a general approach, older and sicker patients with greater surgical risk were considered better suited for percutaneous debulking. This may have introduced selection bias.

Sample size was limited by experience of our single center. There is a risk that observed effects may have been driven by chance findings in few patients in either group (type I statistical error). Encouragingly, findings were consistent across patient groups, and variance was low. Nonetheless, a small sample size may have failed to detect significant differences (type II statistical error). The trend toward three-fold higher one-year mortality (*p* = 0.11) and larger right ventricular size (*p* = 0.09) in patients that underwent percutaneous debulking suggested this may have occurred. The patient data set provided insufficient power to determine relationships with gender, co-morbid conditions, or endocarditis recurrence.

A trend toward a difference in 1 year mortality (*p* = 0.11) between therapies may suggest limited durability of percutaneous debulking. We speculate this may in part be due to sequelae of significant tricuspid regurgitation caused by percutaneous debulking that was not present with surgical valve repair or replacement. Incomplete removal of all infected tissue with percutaneous debulking may have also contributed to this finding. The study was not designed to evaluate these questions, which is a limitation.

The distinction between hospital charges, hospital costs, and actualized reimbursement should be considered when interpreting study findings. Postoperative outpatient antibiotic costs were not included in analyses.

## Conclusions

Percutaneous debulking of tricuspid endocarditis achieved similar outcomes to tricuspid valve surgery and avoided implantation of prosthetic material in patients with active IVDA. No financial difference was observed versus open tricuspid valve surgery. Percutaneous debulking should be considered as a possible alternative to surgery for tricuspid endocarditis in select patients. A trend toward a difference in 1 year mortality between therapies may suggest limited durability of percutaneous debulking.

## Data Availability

At request.

## Abbreviations

IVDA: Intravenous drug abuse
IRB: Institutional review board
LVEF: Left ventricle ejection fraction
RV: Right ventricle
TR: Tricuspid regurgitation
